# The evolution of tremor in Parkinson's Disease: insights from a 4-year longitudinal assessment

**DOI:** 10.1007/s10072-025-08363-9

**Published:** 2025-07-22

**Authors:** Carolina Cutrona, Matteo Costanzo, Giorgio Leodori, Maria Ilenia De Bartolo, Agnese Liguori, Francesco Marchet, Marco Mancuso, Giorgio Vivacqua, Antonella Conte, Giovanni Fabbrini, Alfredo Berardelli, Daniele Belvisi

**Affiliations:** 1 Department of Human Neuroscience, Sapienza University of Rome, Viale Dell’Università 30, Rome, 00185 Italy; 2 Department of Neuroscience, Istituto Superiore Di Sanità, Viale Regina Elena 299, Rome, 00161 Italy; 3 IRCCS Neuromed, Via Atinense 18, Pozzilli, IS 86077 Italy; 4 Department of Experimental Morphology and Microscopy- Integrated Research Center (PRAAB), Biomedico University of Rome, Via A. del Portillo 21, Rome, 00125 Italy; 5 Department of Neurology, Ruggi D’Aragona and S.Giovanni Di Dio Hospital, Via San Leonardo, Salerno, 84131 Italy

**Keywords:** Parkinson’s disease, Tremor, Resting tremor, Action tremor, Clinical course, Longitudinal studies

## Abstract

**Introduction:**

While tremor is considered a cardinal motor sign in patients with Parkinson’s disease (PD), little is known about the evolution of the different types of PD tremor over time.

**Aims:**

Our objectives were to assess the rate of progression of the various types of PD tremor over the disease course and to verify if the presence of different tremors is consistently associated with specific motor and non-motor burdens over time. Finally, we investigated whether the presence of different tremors can predict specific trajectories of disease progression.

**Methods:**

One hundred PD patients were enrolled and 73 completed a 4-year follow up. Clinical evaluations included the administration of standardized PD scales to assess the severity of motor and non-motor manifestations. The occurrence and severity of rest, re-emergent, and action tremors were accurately evaluated at baseline and 4 years later. Adjusted linear regression models were used to assess tremor type’s influence on disease progression.

**Results:**

Tremor occurrence and severity decreased during PD progression, with a more significant reduction in action tremor compared to rest and re-emergent tremor. Patients with rest and re-emergent tremor had milder motor symptoms at baseline and milder motor and non-motor manifestations at follow-up. The presence of rest and re-emergent tremor predicted a lower progression of non-motor symptoms.

**Conclusions:**

This longitudinal study revealed that the various types of PD tremor have different evolutions over disease course, can predict distinct trajectories of disease progression and are underpinned by distinct pathophysiological mechanisms.

**Supplementary Information:**

The online version contains supplementary material available at 10.1007/s10072-025-08363-9.

## Introduction

Patients with Parkinson’s disease (PD) may have different types of upper limb tremor [1, 2]. Typically, tremor is present at rest and disappears when the patient performs a voluntary movement or maintains a posture (rest tremor) [1, 3–5]. In patients with rest tremor, tremor can also re-emerge after a variable delay in reaching and maintaining an upper limb posture (re-emergent tremor) [6, 7]. Tremor can appear only after achieving and maintaining a posture (postural tremor) or when patients perform a voluntary movement (kinetic tremor) [8]. Rest and re-emergent tremor share similar pathophysiological [9] and clinical features [6, 7, 10, 11]; they are both characterized by the emergence of tremor under stable motor conditions (“stability tremor”), as opposed to “action tremor”, which includes pure postural and kinetic tremor [1]. Action tremor differs from rest and re-emergent tremor in terms of amplitude [8], frequency, [8, 12] pattern of muscle activation [8], and response to dopaminergic treatment [2, 8, 11]. Furthermore, in patients with action tremor, bradykinetic-rigid features are worse than those present in patients with rest and re-emergent tremor [7, 11, 13]. Previous evidence has suggested that tremor occurrence tends to diminish during the disease course and, unlike other motor signs, tremor severity may even improve as the disease progresses [14]. However, most studies investigating different types of PD tremor have had a cross-sectional design, and little is known about the rate of progression and degree of consistency of the different PD tremors over time. Only two longitudinal studies assessed the progression of the different types of PD tremor; however, none of these two studies evaluated re-emergent tremor [13, 15], now considered the most frequent tremor in PD after rest tremor [8].

In this longitudinal 4-year follow-up study, we aimed to assess in PD i) whether the different types of tremor remain stable or modify their clinical features over time, by evaluating tremor occurrence and severity of tremor with a 4-year follow-up; ii) whether the clinical differences of motor and non-motor symptoms between patients with different types of tremor remain constant or change during disease progression; iii) whether the different PD tremor can predict specific trajectories of disease progression in terms of severity of motor and non-motor symptoms.

## Materials and methods

### Subjects

A total of 100 Parkinson's Disease (PD) patients (40 women and 60 men) were consecutively enrolled at the outpatient Movement Disorder clinic of the Department of Human Neurosciences, Sapienza University of Rome, and IRCSS Neuromed. The inclusion criteria were: i) patients with a diagnosis of PD according to the international diagnostic criteria; ii) age over 18 years; iii) the ability to provide written informed consent for participation in the study. The exclusion criteria were: i) a diagnosis of atypical parkinsonism; ii) a diagnosis of secondary and/or iatrogenic parkinsonism; iii) inability to sign the informed consent. We collected demographic and clinical data included age, age at disease onset, disease duration, duration of dopaminergic drug treatment, Levodopa Equivalent Daily Dose (LEDD), presence of dyskinesia or motor fluctuations, comorbidities and non dopaminergic treatment. The study received approval from the Sapienza University of Rome ethics committee (n. 4734) and complied with the Declaration of Helsinki. All participants provided written informed consent.

### Clinical evaluation

Disease severity and motor symptoms were assessed using the Hoehn and Yahr scale (H&Y) and the Movement Disorder Society-sponsored revision of the Unified Parkinson’s Disease Rating Scale (MDS-UPDRS) part III. Non-motor symptoms were evaluated with the Non-Motor Symptoms Assessment Scale for PD (NMSS). The levodopa-equivalent daily dose (LEDD) was also calculated. To assess the weight of comorbidities we calculated the Charlson Comorbidity Index (CCI).

Rest tremor was assessed with patients sitting comfortably on a chair with upper limbs at rest for 60 s. Re-emergent and postural tremor were evaluated by having patients extend their arms for 90 s. Kinetic tremor was assessed using finger-to-nose and finger-to-finger tests (15 times per side for each test). Re-emergent tremor was defined as tremor reappearing after a variable delay after reaching and mantaining a posture in PD patients with resting tremor. The severity of re-emergent tremor was scored 0–4 according to the MDS-UPDRS item for tremor. Body distribution of re-emergent tremor was registered. All patients with tremor exhibited rest tremor alone or with re-emergent or action tremor. Patients were evaluated off treatment.

### Statistical analysis

For the statistical analysis, we used SPSS software (SPSS Inc., Chicago, IL, USA) version 25.

Descriptive analysis techniques were used for demographic and clinical data, including baseline tremor and the 4-year follow-up evaluation.

A paired T-test was used to compare tremor severity at the baseline and the 4-year follow-up evaluation. Then, we compared the clinical features of patients with different types of tremors with those without tremor. For this purpose, we considered three groups: patients with rest tremor alone or in combination with re-emergent tremor, patients with resting tremor associated with action tremor, and patients without any type of tremor. The analysis was performed only in patients with the same tremor subtype at the baseline and at the 4-year follow-up evaluation. Through the Kruskal–Wallis’s test, the disease stage (H&Y scale score), the severity of motor and (MDS-UPDRS III score), non-motor symptoms (NMSS score), and dopaminergic therapy (expressed in LEDDs) were compared. Mann–Whitney U test was used for the post hoc analysis. FDR was used to correct for multiple comparisons. P values < 0.05 were considered as significant.

Adjusted linear regression models were designed to assess whether the presence of different types of tremors at the baseline could influence disease progression. In these models, the dependent variables included the progression of the disease stage (expressed as the H&Y scale score ratio), the severity of motor symptoms (MDS-UPDRS III score ratio), the severity of non-motor symptoms (NMSS score), and dopaminergic therapy (expressed in LEDDs ratio). Disease progression was evaluated as the ratio of the scores of various clinical scales at follow-up assessment/baseline assessment *100. The type of tremor represented the independent variable (rest tremor alone or associated with re-emergent tremor, action tremor, or no tremor), assessed at the baseline. Age, sex, and disease duration were included as covariates to adjust each regression model, as they were considered relevant factors influencing PD disease progression.

## Results

### Clinical and demographic data at baseline (T0) and follow-up(T1)

One hundred patients were enrolled at the baseline and 73 completed the follow up assessment. Demographic and clinical data of patients are reported in Table 1. At T0, eighty-two patients were either on levodopa alone (39) or on levodopa combined with other drugs (43), 7 patients were on dopamine agonists alone, 5 were on monoamine oxidase inhibitor type B alone, and 6 were on dopamine agonists combined with monoamine oxidase inhibitor type B. At T1, 67patients were on levodopa alone (27) or in combination with other drugs (40), while 2 patients were on monoamine oxidase B inhibitors alone (1) or in combination with dopamine agonists (1). At both time points, the distribution of tremor was closely aligned with the body side most affected by bradykinetic-rigid symptoms (95% at T0 and 98% at T1). The CCI was similar in RT/RET, AT and no tremor groups (*p* > 0.05) (Supplementary Table 1).

**Table 1:** Demographic and clinical data of patients enrolled at T0 (100 patients) and T1 (73 patients)

	T0	T1
Age (years)	69.1 ± 9.6	72.21 ± 8.9
Disease duration (years)	5.7 ± 2.7	8.34 ± 3.8
Female (%)	34%	34%
LEDD (mg)	518.2 ± 211.8	757.9 ± 232.8
MDS-UPDRS III	29.4 ± 7.8	46.58 ± 15.8
H&Y stage	2.1 ± 1.5	2.75 ± 0.6
NMSS score	47.7 ± 32.1	71.4 ± 31.8
Dyskinesias (% of patients)	24	31
Motor fluctuations (% of patients)	46	52

### Longitudinal evaluation of different types of tremors

**Fig. 1:**
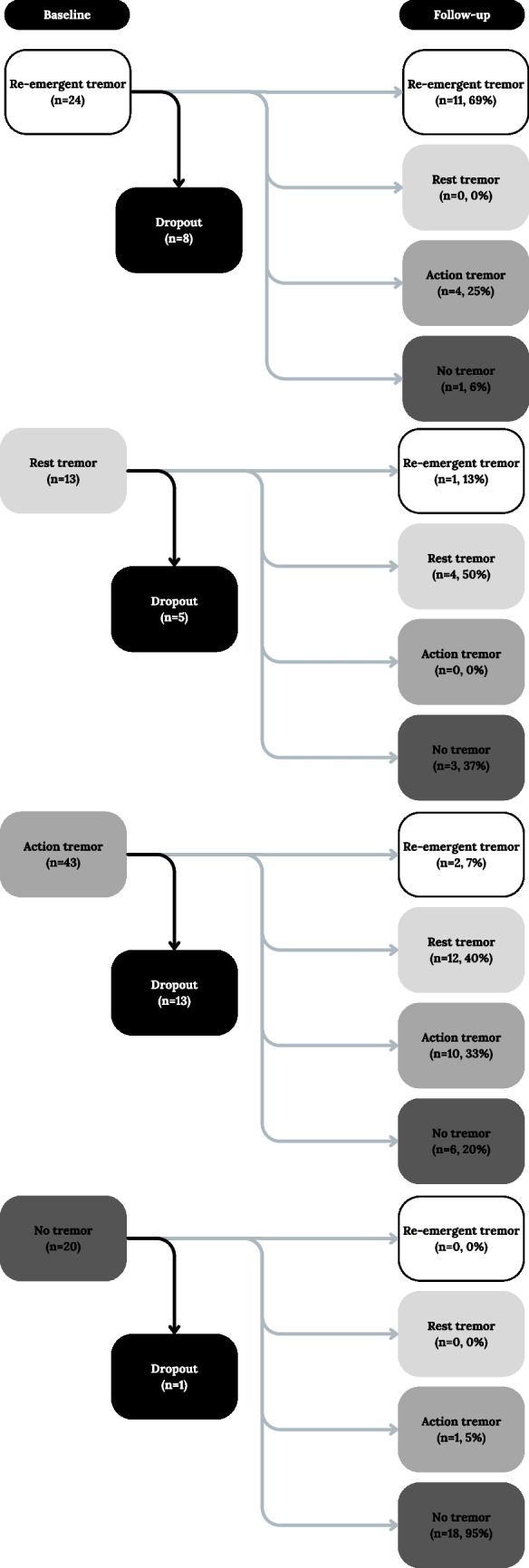
Distribution of PD tremors at T0 and T1

Figure 1 reports the distribution of PD tremors in our population at T0 and T1. In evaluating the course of tremor over time, we found that isolated RT was confirmed in the same patient at follow-up evaluation in 50% (4/8 who completed the follow up), RET was confirmed in the same patients at T1 in 69% (11/16 who completed the follow up), AT was confirmed in the same patients at T1 in 33% (10/30 who completed the follow up), the absence of tremor was confirmed in the same patients at T1 in 95% (18/19 who completed the follow up). In patients who showed the same type of tremor at baseline and at follow-up evaluation, the severity of the tremor was significantly reduced (resting tremor T = 2.66; *p* = 0.01 re-emergent tremor T = 2.69; *p* = 0.02; action tremor T = 2.4; *p* = 0.002).

### Clinical features of patients with and without tremor

At baseline, patients with rest tremor alone or in combination with re-emergent tremor, patients displaying action tremor, and patients with no tremor showed a similar sex distribution, age, age at onset, and disease duration (all *p* values > 0.05). Conversely, the three groups differed in terms of motor symptoms severity (MDS-UPDRS part III score: H = 13.215; *p* = 0.001) and stage of disease (H&Y score: H = 14.134; *p* = 0.001). Non-motor symptom severity (NMSS score: H = 0.407; *p* = 0.816) and LEDDs (H = 0.326; *p* = 0.849) were similar among the three groups. The post hoc analysis showed that patients with rest tremor alone or in combination with re-emergent tremor had lower MDS-UPDRS part III and H&Y score than patients with action tremor (MDS-UPDRS part III score: U = 76; Z = −3.673; *p *= 0.0002; H&Y score: U = 145.5; Z = −2.311; *p *= 0.021) and patients with no tremor (MDS-UPDRS part III score: U = 58.5; Z = −2.101; *p *= 0.035; H&Y score: U = 32.5; Z = −3.625; *p* = 0.001). Patients with action tremor had a lower H&Y score than patients with no tremor (H = 93.5; Z = 2.030; *p* = 0.042) but similar MDS-UPDRS part III score (U = 132.5; Z = -0.569; *p* = 0.569).

The 4-year follow-up evaluation displayed that the three groups differed in terms of MDS-UPDRS part III score (H = 13.032; *p* = 0.001), H&Y score (H = 21.182; *p* = 0.00002) and NMSS score (H = 11.325; *p* = 0.003). LEDDs did not differ among the three groups (H = 6.631; *p* = 0.036 non-significant after correction for multiple comparisons).

The post hoc analysis revealed that patients with rest tremor alone or in combination with re-emergent tremor had lower MDS-UPDRS part III, H&Y, and NMSS scores than both patients with action tremor and no tremor (rest and re-emergent tremor vs. action tremor: MDS-UPDRS part III score: H = 107; Z = −2.911; *p* = 0.004; H&Y score: H = 112; Z = −2.999; *p* = 0.003; NMSS score: U = 115.5; Z = −2.697; *p* = 0.007; rest and re-emergent tremor vs. no tremor: MDS-UPDRS score: U = 31.5; Z = −3.246; *p* = 0.001; H&Y score: U = 13; Z = −4.333;* p* = 0.00001; NMSS score: Z = 36; Z = −3.051; *p *= 0.002).

**Fig. 2:**
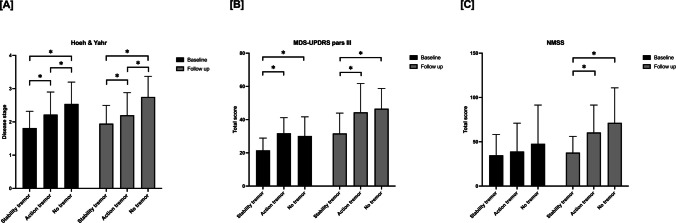
Clinical features of patients with rest tremor alone or in combination with re-emergent tremor, action tremor and no tremor. Comparison of Hoehn and Yahr Scale (**A**), MDS-UPDRS III scale (**B**) and Non-Motor Symptom Scale (**C**) scores among patients with rest tremor alone or combined with re-emergent tremor, action tremor, and no tremor at baseline and follow-up. The * indicates the presence of statistically significant differences

The comparison between patients with action tremor and patients with no tremor showed a significantly higher H&Y score in the first group (H: 78.5; Z = −2.551; *p* = 0.01) with similar MDS-UPDRS part III (H = 122; Z = −0.910; *p* = 0.363) and NMSS score (U = 124.5; Z = −0.828; *p *= 0.408) (See Fig. 2).

### Relationship between tremor and disease progression in terms of motor and non-motor symptoms

**Fig. 3:**
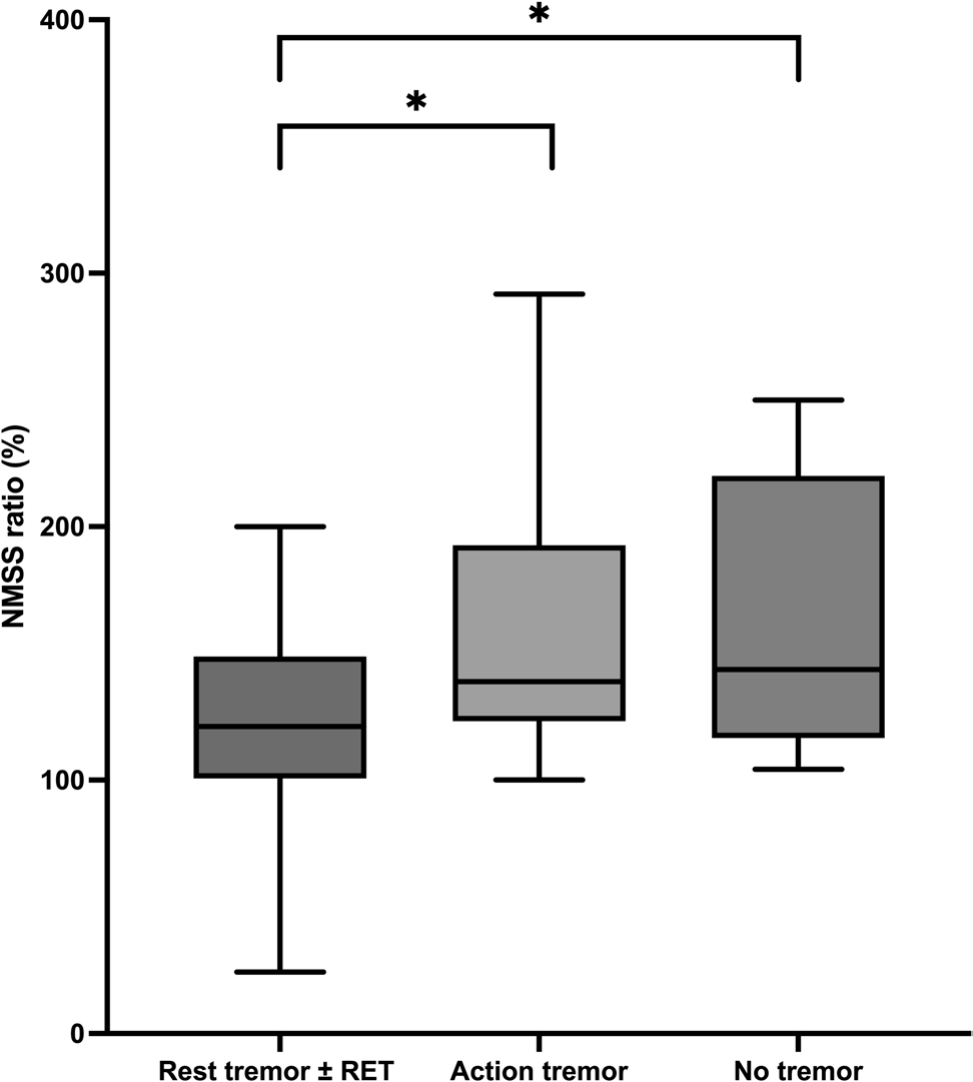
Relationship between tremor and non-motor symptoms progression. Rest tremor alone or combined with re-emergent tremor were associated to lower progression of non-motor symptoms (*NMSS*) compared to non tremulous patients in the adjusted linear regression models. The * indicates the presence of statistically significant differences

The adjusted linear regression models revealed that rest tremor alone or combined with re-emergent tremor was associated with lower progression of non-motor symptoms (B = -77,838; t = −2,071; *p* = 0.043). Conversely, the absence of tremor was associated with a greater progression of non-motor symptoms (B = 133,878; t = −3,036; *p* = 0.004). No association was observed between action tremor and disease progression (B = −13,124; t = −0,314; *p* = 0.755) (See Fig. 3).

## Discussion

The first aim of the present study was to investigate whether the different PD tremors remain stable or change over time by evaluating tremor occurrence and severity with a 4-year follow-up. We found a reduction in the occurrence and severity of the various types of PD tremor throughout the disease, with a more relevant reduction in action tremor compared to rest and re-emergent tremors.

The observation that, in PD, tremor tends to reduce over time is in line with previous evidence showing a significant reduction in the prevalence of tremor-dominant in favor of non-tremor-dominant patients during the disease course [13, 14, 16–19]. Only few authors observed that tremor severity and occurrence remained stable during the disease course [20–22].

Pasquini et al. have recently performed a longitudinal analysis of the PPMI cohort reporting that off-state rest, postural and kinetic tremor scores increased significantly over time during the first 7 years of the disease. The comparison between this study conducted in de novo patient and our and previous studies including patients with variable disease duration [13, 14, 16–19] suggests that the consistently reported reduction of tremor during disease progression could be preceded by an early phase characterized by an increase in tremor severity and prevalence [15].

None of the longitudinal studies that analyzed the progression of tremors throughout disease progression have considered re-emergent tremor as a distinct tremor type [13, 15–18]. Therefore, our study provides the first longitudinal evidence on the evolution over the disease course of rest, re-emergent, and action tremor. The finding that rest tremor and re-emergent tremor exhibit similar behavior throughout the disease's progression agrees with several previous clinical [6–8, 11] and neurophysiological [9, 12] evidence suggesting that rest tremor and re-emergent tremors share similar pathophysiological mechanisms due to the involvement of the dopaminergic system. Conversely, the observation that action tremor is more unstable during disease progression when compared to rest and re-emergent tremors adds a distinguishing element between these PD tremors [7, 9, 11]. Previous findings suggest that AT has a more relevant involvement of serotoninergic and cerebellar pathways when compared with rest tremor and from a pathophysiological point of view seems to reflect the activity of non-dopaminergic systems [19, 20]. Accordingly, independent groups demonstrated that dopaminergic treatment induces a better clinical response in RT [11, 15] and RET [11]than in AT. It is, therefore, plausible that the progressive dysfunction of non-dopaminergic systems during disease course may account for the longitudinal instability of action tremor [19].

In line with previous evidence we reported a co-localization of all types of tremor and bradykinetic-rigid symptoms. To identify the common thread among the pathophysiological mechanisms underlying the various symptoms of PD some authors suggested that tremor onset, produced by the oscillatory activation of the cortico-cerebellar network could be partly triggered by the basal ganglia dysfunction underlying symptoms of rigidity and bradykinesia [21].

The second aim of our study was to determine whether the clinical differences observed (transversally) between patients with rest tremor alone or in combination with re-emergent and action tremor persist or change during disease progression. We first confirmed that motor symptoms are milder in patients with rest tremor alone or in combination with re-emergent tremor than in patients with action tremor [7, 11, 13]. The novel finding of the present longitudinal study is that the clinical differences between patients with different types of tremors are still present over time, becoming even more evident during the disease course. The present findings support the hypothesis that rest tremor, alone or in combination with re-emergent tremor, is a clinical highlight of a benign PD subtype.

The finding that action tremor, when compared to rest and re-emergent tremor, is associated with more severe bradykinetic-rigid symptoms and is more'unstable'during the disease course suggests that action tremor may represent an intermediate phenotype in terms of disease progression between patients with rest/re-emergent tremor and those without tremor, disclosing a prominent bradykinetic-rigid phenotype.

A further aim of the present study was to investigate whether the different PD tremors can predict specific trajectories of disease progression. By applying the adjusted linear regression models, we found that rest tremor alone or combined with re-emergent tremor was associated with a lower progression of non-motor symptoms severity after 4 years of follow-up; in contrast, patients with no tremor had a greater progression of non-motor symptoms. Patients with action tremor did not show significant association with non-motor symptom progression. Several authors have shown that patients with tremor-dominant PD exhibit a relatively slower disease progression [22–25]. However, previous studies have used a combined score of resting and postural/action tremor according to the UPDRS part III to distinguish between tremor-dominant and akinetic-rigid subtypes. Therefore, it remained unclear which type of tremor better predicts clinical benefits associated with the tremor-dominant subtype. Our study provides evidence supporting the hypothesis that rest tremor alone or in combination with re-emergent tremor, differently from action tremor, is characterized by lower nonmotor symptom progression when compared to non-tremulous patients and is a stronger predictor of non-motor symptom progression in PD.

Overall, our findings suggest that distinct pathophysiological mechanisms underlie the progression of the various types of PD tremor. This hypothesis needs to be validated by the identification of tremor-specific biomarkers able to distinguish between different types of PD tremor. Future molecular, neurophysiological and neuroimaging investigations are needed to better define the pathological substrate of different tremors evolution in patients with PD.

The present study is not free from some limitations. Although we performed an off-treatment evaluation at both baseline and follow-up clinical assessment, we cannot fully exclude possible long-term confounding effects of pharmacological treatment on tremor and motor symptoms scores. In particular, we cannot fully exclude that levo-dopa long-duration response (LDR) effects could have influence milder motor manifestations during the clinical assessment. However, given the relatively long disease duration at the baseline we believe that LDR confounding effects could have a negligible effect on our findings. A further limitation of our study was that we included patients with different disease duration and we did not have information on tremor type at disease onset. Therefore, future studies investigating de novo patients at early phase are needed. Moreover, since our assessment was purely clinical, we cannot exclude that some patients classified as postural tremor had a re-emergent tremor with a very short appearance latency.

## Conclusions

The present longitudinal description of tremor progression in PD showed a reduced occurrence of tremor during the disease course. The similar rate of progression between rest and re-emergent tremors represents the first longitudinal evidence of the interconnection between these types of tremor. The evidence that rest tremor alone or in combination with re-emergent tremor is associated with milder severity of motor and non-motor symptoms over time and associated with a slower non-motor symptom progression supports its relevant weight in defining the benignity of tremulous subtype in PD.

## Supplementary Information

Below is the link to the electronic supplementary material.

Supplementary file1 (PDF 96.3 KB)

## Data Availability

The data supporting the findings of this study are available on request from the corresponding author. The data are not publicly available due to privacy or ethical restrictions.
