# Supplementary material for: The evolution of tremor in Parkinson's Disease: insights from a 4-year longitudinal assessment
Source: Neurol Sci. 2025 Jul 22;46(9):4397–403. doi: 10.1007/s10072-025-08363-9 (PMC12394333; doi:10.1007/s10072-025-08363-9)
Supplement: Supplementary file 1 — Supplementary file1 (PDF 96.3 KB) [file 10072_2025_8363_MOESM1_ESM.pdf]

Patient ID	Comorbidities	Charlson index (CCI)	Non dopaminergic treatment
**RET1**	labyrinthitis, arterial hypertension	4	antihypertensive therapy
**RET2**	none	3	none
**RET3**	cancer	9	Tamoxifene
**RET4**	benign prostatic hyperplasia, arterial hypertension, vasculopathy	5	5 alpha reductase inhibitors
**RET5**	diabetes mellitus	5	oral antidiabetics
**RET6**	n.a.	n.a.	n.a.
**RET7**	arterial hypertension	3	antihypertensive therapy
**RET8**	mood disorder	4	antidepressant drugs
**RET9**	arterial hypertension	4	antihypertensive therapy
**RET10**	hip prosthesis	4	FANS
**RET11**	post-traumatic bone fractures	3	none
**RET12**	inguinal hernia	3	none
**RET13**	none	4	none
**RET14**	peptic ulcer	4	proton pump inhibitors
**RET15**	TBC, Mood disorder, cancer	5	BDZ
**RET16**	prostate cancer, pulmonary hamartoma, arterial hypertension	7	antihypertensive therapy
**RET17**	hodgkin's lymphoma, arterial hypertension, Ischaemic heart disease, diabetes mellitus	7	Chemotherapy, beta blockers, oral antidiabetics, antiplatelet therapy
**RET18**	Dyslipidemia	4	none
**RET19**	arterial hypertension, atrial fibrillation, dyslipidemia, left anterior hemiblock	4	beta blockers, antihypertensive therapy, antiplatelet therapy, statins
**RET20**	none	3	none
**RET21**	Ischaemic heart disease, arterial hypertension, bilateral cataract	5	BDZ, antiplatelet therapy, beta blockers, anticoagulants, proton pump inhibitors
**RET22**	arterial hypertension, glaucoma, MGUS, Gallstones	3	antihypertensive therapy
**RET23**	none	4	none
**RET24**	hypothyroidism, osteoporosis	3	thyroid hormones
**RT1**	none	2	none
**RT2**	hypertrophic heart disease, arterial hypertension, Retinopathy	5	antiplatelet therapy
**RT3**	none	3	none
**RT4**	arterial hypertension, benign prostatic hyperplasia	3	antihypertensive therapy
**RT5**	benign prostatic hyperplasia, perianal fistula	4	none
**RT6**	n.a.	n.a.	n.a.
**RT7**	Hypothiroidism, migraine	4	thyroid hormones
**RT8**	Gallstones,arterial hypertension, Gastritis, Osteoporosis, Mood disorder	4	antidepressant drugs, Zdrugs, antihypertensive therapy

**RT9**	cancer	9	chemotherapy
**RT10**	mood disorder	4	antidepressant drugs
**RT11**	carpal tunnel, cancer	2	chemotherapy
**RT12**	Ischaemic heart disease, heart failure, cancer, benign prostatic hyperplasia, dyslipidemia, arterial hypertension	8	proton pump inhibitors, antihypertensive therapy, antiplatelet therapy, statins, Vitamine D
**RT13**	Hypothyroidism, arterial hypertension, dyslipidemia	3	thyroid hormones, antiplatelet therapy, statins
**AT1**	arterial hypertension, vasculopathy	5	antiplatelet therapy
**AT2**	Appendicectomy	4	none
**AT3**	diabetes mellitus, Hernia inguinale	6	oral antidiabetics
**AT4**	arterial hypertension, vasculopathy	5	antihypertensive therapy
**AT5**	cancer, benign prostatic hyperplasia, allergic rhinitis	4	5 alpha reductase inhibitors
**AT6**	none	3	none
**AT7**	Atrial fibrillation, dyslipidemia	4	anticoagulant drugs, beta blockers,statins
**AT8**	Migraine	1	none
**AT9**	benign prostatic hyperplasia, diabetes mellitus, arterial hypertension, Dyslipidemia	7	Oral antidiabetics, statins, antihypertensive therapy,alphalytic therapy
**AT10**	cancer, arterial hypertension, dyslipidemia, benign prostatic hyperplasia	10	Finasteride, Ranitidina, Antiplatelet, antihypertensive therapy, statins
**AT11**	none	4	none
**AT12**	mood disorder	4	antidepressant drugs
**AT13**	nodular thyroid disease, osteoporosis, mood disorder, cancer	9	Tamoxifene
**AT14**	none	2	none
**AT15**	arterial hypertension,hearing loss, nephrolithiasis	4	antihypertensive therapy
**AT16**	gallstones, uterine fibroid,dyslipidemia,arterial hypertension	4	antihypertensive therapy, antiplatelet therapy
**AT17**	arterial hypertension, atrial fibrillation, dyslipidemia, depression	4	antidepressant drugs, anticoagulant drugs; beta blockers; thyroid hormones, antihypertensive therapy, Omega3
**AT18**	arterial hypertension, dyslipidemia	4	antihypertensive therapy
**AT19**	arterial hypertension	3	antihypertensive therapy
**AT20**	Appendicectomia, dyslipidemia, mood disorder	3	statins, antidepressant drugs
**AT21**	acoustic neuroma	4	none
**AT22**	Atrial fibrillation, arterial hypertension	4	antihypertensive therapy, antiarrhythmics
**AT23**	none	3	none
**AT24**	mood disorder	3	antidepressant drugs

**AT25**	arterial hypertension, ischaemic heart disease, dyslipidemia	6	antiepileptics; antiarrhythmics, Statins, antihypertensive therapy, antiplatelet therapy
**AT26**	none	2	none
**AT27**	arterial hypertension, carotid atheroma, atrioventricular block	5	antiplatelet therapy, Statins
**AT28**	diabetes mellitus	5	oral antidiabetics
**AT29**	arterial hypertension, COPD	3	antihypertensive therapy, corticosteroids
**AT30**	liver cirrhosis, benign prostatic hyperplasia	7	proton pump inhibitors, Levolac, 5 alpha reductase inhibitors, antihypertensive therapy
**AT31**	arterial hypertension, Thyreopathy, carpal tunnel, gastritis	4	proton pump inhibitors, thyroid hormones
**AT32**	ovarian cyst, Gallstones,arterial hypertension, Osteoporosis	4	beta blockers, antihypertensive therapy
**AT33**	venous insufficiency, rectal prolapse, arterial hypertension,dyslipidemia	6	proton pump inhibitors, antihypertensive therapy, Omega3, Allopurinol, statins
**AT34**	post-surgical hypothyroidism,gallstones,arterial hypertension	4	thyroid hormones, Cabergoline,antihypertensive therapy
**AT35**	hypothyroidism, bilateral blepharoplasty	2	thyroid hormones
**AT36**	none	4	none
**AT37**	Uterine fibroid,arterial hypertension,mood disorder	4	beta blockers, antihypertensive therapy, antidepressant
**AT38**	gallstones, arterial hypertension, dyslipidemia	3	statins, antihypertensive therapy
**AT39**	nephrolithiasis, arterial hypertension, hyperthyroidism, duodenal ulcer	4	antihypertensive therapy, proton pump inhibitors
**AT40**	none	2	none
**AT41**	arterial hypertension	4	antihypertensive therapy
**AT42**	mood disorder	3	antidepressant drugs, BDZ
**AT43**	benign prostatic hyperplasia, aortic dissection	4	antiplatelet therapy, folic acid, antihypertensive therapy; 5 alpha reductase inhibitors
**NT1**	Retinopathy, benign prostatic hyperplasia	4	alphalytic therapy
**NT2**	uterine fibroid, hip prosthesis, chron disease, depression	4	antidepressant drugs, BDZ
**NT3**	arterial hypertension, vasculopathy	5	antiplatelet therapy
**NT4**	Hashimoto thyroiditis, dyslipidemia, vasculopathy	5	antiplatelet therapy, thyroid hormones
**NT5**	none	3	none
**NT6**	bilateral cataract	3	none
**NT7**	arthrosis	4	coxib
**NT8**	benign prostatic hyperplasia	2	alphalytic therapy
**NT9**	none	4	none
**NT10**	bipolar disorder, cancer , arterial hypertension, diabetes mellitus	7	antiepileptics, antidepressant drugs, antihypertensive therapy, oral antidiabetics

**NT11**	mood disorder	4	antidepressant drugs
**NT12**	appendicectomia, gallstones, benign prostatic hyperplasia, renal cysts, chronic lumbago	4	alphalytic therapy
**NT13**	benign prostatic hyperplasia	3	alphalytic therapy
**NT14**	lombosciatalgia	3	none
**NT15**	Sydenham Chorea, arterial hypertension, mood disorder, dementia	5	antihypertensive therapy, SSRI
**NT16**	mood disorder	4	BDZ
**NT17**	arterial hypertension, cardiopathy, hyperthyroidism, bradycardy (PMK)	5	tapazole, antihypertensive therapy
**NT18**	diabetes mellitus, chronic renal failure, dyslipidemia, drug-induced pancreatitis	8	statins, antidiabetic oral drugs
**NT19**	arterial hypertension,post-surgical hypothyroidism	4	antihypertensive therapy, thyroid hormones
**NT20**	MGUS, cancer	10	antidepressant drugs, chemotherapy

Supplementary Table 1: Comorbidities, Charlson Index and non-dopaminergic drugs of the patients enrolled in our sample. N.a.: not available
